# Overexpressed miR‐486 in bone marrow mesenchymal stem cells represses urethral fibrosis and targets Col13a1 in urethral stricture rats

**DOI:** 10.1002/ccs3.12028

**Published:** 2024-04-22

**Authors:** Yali Xu, Lihong Huang, Zhixin Qiu, Jiaqi Zhang, Xueyi Xue, Junshan Lin

**Affiliations:** ^1^ Department of Pediatric Surgery The First Affiliated Hospital Fujian Medical University Fuzhou China; ^2^ The First Clinical Medical School Fujian Medical University Fuzhou China; ^3^ Department of Urology The First Affiliated Hospital Fujian Medical University Fuzhou China

**Keywords:** BMSCs, Col13a1, fibrosis, miR‐486, urethral stricture

## Abstract

Urethral stricture (US) is a challenging problem in urology and its pathogenesis of US is closely related to the fibrotic process. Previous evidence has indicated the downregulation of microRNA (miR)‐486 in injured urethral specimens of rats. This study aimed to explore the effects of miR‐486‐overexpressed bone marrow mesenchymal stem cells (BMSCs) on US. BMSCs were identified by detecting their multipotency and surface antigens. Lentivirus virus expressing miR‐486 was transduced into rat BMSCs to overexpress miR‐486. Transforming growth factor (TGF)‐β1 induced fibrotic phenotypes in urethral fibroblasts (UFs) and rat models. Western blotting showed protein levels of collagen I/III and collagen type XIII alpha 1 chain (Col13a1). Real time quantitative polymerase chain reaction was utilized for messenger RNA level evaluation. Hematoxylin‐eosin, Masson's trichrome, and Von Willebrand Factor staining were conducted for histopathological analysis. Immunofluorescence staining was employed for detecting alpha smooth muscle actin (α‐SMA) expression. Luciferase reporter assay verified the interaction between miR‐486 and Col13a1. The results showed that miR‐486‐overexpressed BMSCs suppressed collagen I/III and α‐SMA expression in TGF‐β1‐stimulated UFs. miR‐486‐overexpressed BMSCs alleviated urethral fibrosis, collagen deposition, and epithelial injury in the urethral tissue of US rats. miR‐486 targeted and negatively regulated Col13a1 in US rats. In conclusion, overexpression of miR‐486 in BMSCs targets Col13a1 and attenuates urethral fibrosis in TGF‐β1‐triggered UFs and US rats.

## Graphical abstract

Overexpression of miR‐486 in BMSCs targets Col13a1 in urethral fibroblasts and suppresses collagen I/III and α‐SMA expression fibroblasts, thereby suppressing TGF‐β1‐induced urethral fibrosis.

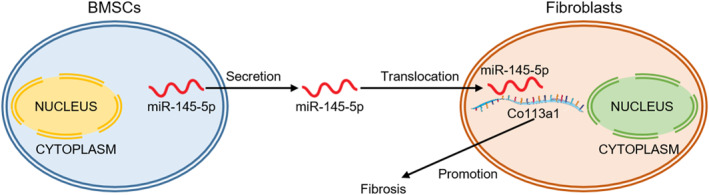

## 1 INTRODUCTION

Urethral stricture (US) is a common clinical disease in urology referring to narrowing of the urethra.^1^ US is featured with fibrosis of tissues surrounding the urethral lumen and results in obstructive symptoms, which adversely affect patients' quality of life.^2^ The etiology of US can be divided into four categories: trauma, inflammation, idiopathic and iatrogenic injury, among which trauma and iatrogenic injury are the most common causes of US in China.^3^ With the development of modern techniques, various treatment options are available for US such as urethral dilatation, internal urethrotomy, and open urethroplasty.^4^ Urethroplasty is considered the gold standard of US treatment, with higher success rates than dilatation or urethrotomy.^5^ However, urethroplasty is still associated with a high recurrence rate even at certain experienced centers.^6^, ^7^ Thus, the management of US is still a clinical challenge.

The pathology of US is closely associated with the fibrotic process, which is characterized by excessive fibroblast proliferation and deposition of extracellular collagen, especially collagen I and III.^8^ Transform growth factor beta1 (TGF‐β1) is a multifunctional polypeptide cytokine belonging to the TGF‐β superfamily of proteins.^9^ TGF‐β1 is widely recognized as a key driver of fibrosis, which can induce collagen production in fibroblasts and consequently trigger fibrotic diseases, including US.^10^ In addition, it was indicated that subcutaneous injection of TGF‐β1 significantly promoted collagen deposition and a fibrotic tissue response.^11^ Thus, TGF‐β1 was used in this study to induce US in fibroblasts as well as in animals.

Bone marrow mesenchymal stem cells (BMSCs) are a group of multipotent cells with self‐renewal ability and multipotency.^12^ Mounting evidences have suggested that BMSCs may serve as promising therapeutic agents in various disorders.^13^ Transplantation of BMSCs has been indicated to exert an alleviative effect on multiple fibrotic diseases, such as hepatic and renal fibrosis.^14^, ^15^ Importantly, Shi et al. demonstrated that BMSCs could prevent the formation of US via the paracrine mechanism in a rat model.^16^ Nonetheless, due to the unfavorable microenvironment, such as inflammation at the damaged site, the viability of mesenchymal stem cells (MSCs) at the transplanted site may be limited, thereby decreasing their potential therapeutic performance.^17^ Recent studies have tried to augment the therapeutic effects of MSCs via exogenous stimulation or endogenous genetical modification.^18^ Particularly, emerging evidences have indicated that microRNAs (miRNAs)‐based genetic modification of MSCs shows great potential in regenerative medicine. For instance, Fu et al. proposed that overexpression of miR‐21 in BMSCs ameliorates chemotherapy‐triggered ovarian injury and improves ovarian function.^19^ Similarly, upregulation of microRNA‐202‐3p in BMSCs alleviates cerebral ischemia‐reperfusion injury by modulating TLR4‐mediated inflammation.^20^

miRNAs are small endogenous nonprotein‐coding RNAs with 19–22 nucleotides that regulate gene expression at the post‐transcription.^21^ They can interact with the 3′ untranslated regions (3′UTRs) of downstream target messenger RNAs (mRNAs), consequently destabilizing mRNAs or repressing translation.^22^ Several miRNAs have been indicated to participate in regulating the progression of US, such as miR‐146a, miR‐4691‐5p, and let‐7i‐5p.^4^, ^23^, ^24^ Intriguingly, a recent study demonstrated that miR‐486 was prominently downregulated after urethral injury.^25^ Nonetheless, it is unclarified whether miR‐486 has an effect on the development of US.

Herein, we aimed to examine the effects of miR‐486‐overexpressed BMSCs on TGF‐β1‐induced US. The molecular mechanism of miR‐486 was also under investigation. It was speculated that overexpression of miR‐486 in BMSCs might affect TGF‐β1‐triggered urethral fibrosis by targeting downstream mRNAs.

## 2 MATERIALS AND METHODS

### 2.1 Culture, identification, and transfection of BMSCs

Primary rat BMSCs were obtained from Procell and cultured in the complete medium (Procell) at 37°C in a humidified atmosphere with 5% CO_2_. The culture medium was changed every 2 days until they reached approximately 80% confluence. Cells at passage 3 were used for subsequent experiments.

To identify the multilineage differentiation potential of BMSCs, the cells were incubated with osteogenic, adipogenic, and chondrogenic differentiation media (Procell), followed by staining with Alizarin Red S, Oil Red O and Alcian blue (Solarbio), respectively. To detect the cell surface markers of BMSCs, the cells were incubated with CD34‐PE, CD44‐FITC, CD90‐FITC, and CD11b‐PE antibodies. The expression of these markers was determined using flow cytometry.

For stable overexpression of miR‐486 in BMSCs, lentivirus vectors (GV309, green fluorescence protein GFP‐labeled) carrying miR‐486 (LV‐miR‐486) were synthesized by GeneChem and transduced into BMSCs. The empty GV309 vector not expressing miR‐486 served as a negative control (LV‐NC).

### 2.2 Rats

Male Sprague Dawley (SD) rats (8–9 weeks, 260–320 g) were purchased from Cavens Experimental Animal Center and housed under standard conditions (temperature 23 ± 2°C, humidity 50%–65%, 12‐h light/dark cycle) with free access to food and water. The animals were allowed for 1‐week acclimation before the experiments. All animal experiments were conducted following the NIH Guide for the Care and Use of Laboratory Animals and approved by the Ethics Committee of The First Affiliated Hospital, Fujian Medical University. Each effort was made to minimize animals' suffering.

### 2.3 Isolation and culture of UFs

Primary urethral fibroblasts (UFs) were isolated from the urethral submucosa tissues of SD rats as previously described.^26^ Briefly, the urethral mucosa tissues were isolated, washed in phosphate‐buffered saline (PBS), cut into 1‐mm^3^ pieces, seeded in the culture flask and maintained at 37°C in a humidified incubator with 5% CO_2_ for 4 h. After full adherence, the culture flask was added with 5 mL of Dulbecco's modified Eagle's medium (Gibco) supplemented with 10% fetal bovine serum (Gibco). The culture medium was changed every 3 days. When the cells reached 80% confluency, they were isolated by trypsinization and further passaged. The UFs at passages 3–5 were used for subsequent experiments.

### 2.4 Cell coculture

A Transwell system (0.4 μm pore size, Corning Inc.) was used for cell coculture. BMSCs were inoculated in the upper chamber, and UFs were seeded into the lower chamber in the presence of 10 ng/mL TGF‐β1. After 24 h, UFs were collected for further analysis.

### 2.5 Luciferase reporter assay

TargetScan database (http://www.targetscan.org/vert_71/) predicts the putative binding site between miR‐486 and collagen type XIII alpha 1 chain (Col13a1). Wild type (Wt) or mutant (Mut) sequences of Col13a1 3′UTR containing miR‐486 binding site were subcloned into pmirGLO vector (Promega) to establish pmirGLO‐Col13a1 3′UTR‐Wt/Mut. These constructs were then co‐transfected into UFs with LV‐NC or LV‐miR‐486 using Lipofectamine 3000 (Invitrogen). After 48 h, the luciferase activity was examined using a Dual‐Luciferase Reporter Assay System (Promega).

### 2.6 Animal models

Forty SD rats were randomly grouped as: (1) sham group; (2) US group; (3) US + BMSC‐NC group; and (4) US + BMSC‐miR‐486 group, with 10 rats per group. The rat US model was established according to the previous description.^7^ Rats were anaesthetized by intraperitoneal injection of 3% pentobarbital sodium (2 mL/kg). A PE‐90 urethral catheter was gently inserted into the urethra to facilitate urethra exposure and prevent urethral injury, and then the ventral penile skin was incised to expose the urethra. Next, the rats were injected into the urethral wall with 100 μL of saline containing 1 μg human recombinant TGF‐β1 (Abcam). Five minutes later, a 23G needle was used to make four partial incisions to the urethral wall through all layers until the catheter was visualized. Rats in the sham group received an injection of 100 μL of saline alone. The catheter was then removed, and the penile skin was sutured with 5‐0 absorbable sutures. On the following day after TGF‐β1 injection, rats in the US + BMSC‐NC group and US + BMSC‐miR‐486 group were injected with LV‐NC‐ or LV‐miR‐486‐transduced BMSCs (BMSC‐NC or BMSC‐miR‐486, 1 × 10^6^/200 μL in saline) into the urethral wall at four different sites (semi‐circumferentially along 1.0 cm of the exposed urethra).

### 2.7 In vivo functional evaluation

Four weeks later, urodynamic parameters were evaluated as previously described.^27^, ^28^ Briefly, rats were anaesthetized, and a polyethylene catheter was implanted in the bladder dome through the urethra. The catheter was connected to the urodynamic testing machine and infusion pump via a T‐tube. Three days later, the conscious rats underwent cystometry to evaluate micturition volume and flow pressure.

### 2.8 Sample collection

After cystometry, all rats were sacrificed by cervical dislocation under anesthesia. Then, the penile skin was incised and the periurethral tissue was removed. The penile midshaft tissues were harvested for further analysis.

### 2.9 Histological analysis

Fresh rat penile midshaft tissues were fixed in 4% paraformaldehyde (PFA), paraffin‐embedded, and sliced (5‐μm‐thick). After dewaxing and rehydration, the tissue sections were subjected to hematoxylin‐eosin (H&E) staining and Masson's trichrome staining (Solarbio) following the protocols of the manufacturer. Next, the sections were dehydrated in ethanol, transparent in xylene, and sealed with resinene. The stained tissues were observed under a light microscope (Nikon). For quantitative analysis of Masson's trichrome staining, the results were evaluated by two investigators blinded to animal treatments and scored based on the following criteria: 0 point, absence of staining and fibrosis; 1 point, slight staining and fibrosis (˂25%); 2 point, moderate staining and fibrosis (25%–50%); and 3 point, strong staining and severe fibrosis (˃50%).^29^

### 2.10 IF staining

For immunofluorescence (IF) staining of UFs, cells were fixed in 4% PFA in PBS for 10 min, permeabilized with 0.1% Triton X‐100 for 5 min, and then blocked with 10% normal goat serum for 1 h. Then UFs were incubated overnight at 4°C with anti‐alpha smooth muscle actin (α‐SMA) primary antibody (ab124964, 1:500, Abcam). For IF staining of tissues, rat urethral tissue sections were deparaffined and rehydrated. Heat‐mediated antigen retrieval was performed using Tris‐EDTA buffer pH 9. The sections were then incubated with anti‐α‐SMA (ab124964, 1:500, Abcam) or anti‐Von Willebrand Factor (vWF; ab6994, 1:500, Abcam) primary antibodies at 4°C overnight. Next, the cells or tissues were incubated with Goat Anti‐Rabbit IgG H&L (Alexa Fluor® 488) preadsorbed secondary antibody (ab150081, 1:200, Abcam) at room temperature for 1 h. 2‐(4‐Amidinophenyl)‐6‐indolecarbamidine dihydrochloride (DAPI; Solarbio) was employed for nuclear labeling. The slides were observed under a fluorescence microscope (Nikon), and the results were analyzed by ImageJ software.

### 2.11 Real time quantitative polymerase chain reaction

Total RNA extraction from rat UFs or penile midshaft tissues was performed using TRIzol reagent (Invitrogen). The iScript cDNA Kit (Bio‐Rad) was employed for cDNA preparation. Real time quantitative polymerase chain reaction (RT‐qPCR) was conducted with SYBR Premix Ex TaqTM (Takara) using a StepOnePlus Real‐Time PCR system (Applied Biosystems). Normalized to U6 or glyceraldehyde‐3‐phosphate dehydrogenase (GAPDH), the relative expression of miR‐486 and mRNAs was evaluated using the 2^−ΔΔCt^ method. Primer sequences are listed in Table 1.

**TABLE 1:** Primers used for RT‐qPCR.

Gene	Sequence (5′‐3′)	
miR‐486	Forward	GCGTCCTGTACTGAGCTGC
	Reverse	CGGCCCAGTGTTCAGACTAC
Synm	Forward	GGAAGGAGAAAGCAATCCG
	Reverse	TAGGATGTGTTTCTGGACTCTC
Rbpj	Forward	GACTTGTGCATTGCCTCAG
	Reverse	TCCTTCTACCAGGTATCTGGT
Col13a1	Forward	CTCTAGAGGGCCTAAAGGG
	Reverse	TAAGCCATCTTCTCCCAGTG
Snrpd1	Forward	TTGAGTCATGAAACCGTAACC
	Reverse	CATGCTGACATCTACACCTG
U6	Forward	CAGTTATGACGACCTAGACAG
	Reverse	CAAATTTGCATGTCATCCTTGG
GAPDH	Forward	AACTCCCATTCTTCCACCT
	Reverse	TTGTCATACCAGGAAATGAGC

Abbreviation: GAPDH, glyceraldehyde‐3‐phosphate dehydrogenase.

### 2.12 Western blotting

Protein isolation from rat UFs or penile midshaft tissues was conducted using radio immunoprecipitation assay buffer (Solarbio). Protein concentration was quantified with a bicinchoninic acid assay kit (Beyotime). Protein samples (20 μg) were separated by 10% sodium dodecyl sulfate‐polyacrylamide gel electrophoresis and blotted onto polyvinylidene fluoride membranes (Beyotime). After blocking with 5% defatted milk, the membranes were incubated at 4°C overnight with primary antibodies as follows: anti‐Col13a1, anti‐collagen I, anti‐collagen III, and GAPDH (all from Abcam) followed by incubating with the HRP‐conjugated goat anti‐rabbit secondary antibody (ab7090, Abcam) at room temperature for 2 h. Lastly, blot signaling was visualized using an enhanced chemiluminescence kit (Solarbio), and ImageJ software (NIH, Bethesda, MD) was utilized for relative protein expression quantitation.

### 2.13 Detection of transplanted BMSCs in the urethra

To detect the distribution of GFP‐labeled BMSCs in rat urethra, the penile midshaft tissues were taken for frozen sectioning. Tissue sections were washed three times in PBS, fixed with 4% PFA, permeabilized with 0.2% Triton‐X‐100, and then blocked with 0.02% bovine serum albumin. Afterward, the sections were incubated with anti‐GFP primary antibody (ab183734, 1:500, Abcam) at 4°C overnight and washed in PBS before incubation with the Goat Anti‐Rabbit IgG H&L (Alexa Fluor® 488) secondary antibody (ab150081, 1 μg/mL, Abcam) for 1 h at room temperature. Nuclear DNA was labeled with DAPI. The results were observed under a fluorescence microscope (Nikon).

### 2.14 Statistical analysis

Data are presented as the mean ± standard deviation. Each experiment was repeated at least three times independently. Student's *t*‐test or one‐way ANOVA followed by Tukey's *post hoc* analysis were used for difference comparisons using SPSS 25.0 software (IBM). Pearson's correlation analysis was used to evaluate the expression correlation between miR‐486 and Col13a1. *p*˂0.05 indicated statistical significance.

## 3 RESULTS

### 3.1 Identification of BMSCs

**FIGURE 1:**
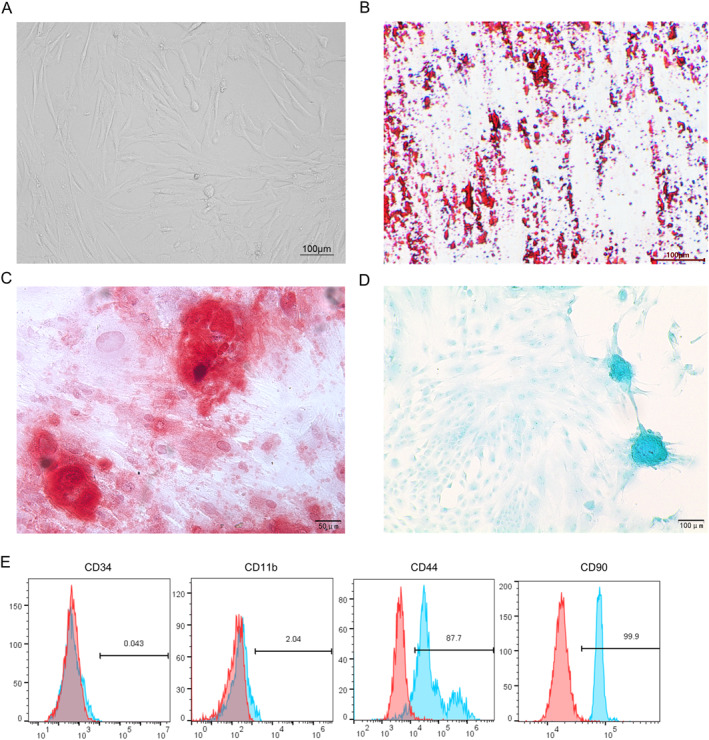
Identification of BMSCs. (A) Morphology of BMSCs at passage 3. (B–D) Representative images of Oil red O staining (B), Alizarin red S staining (C) and Alcian blue staining (D) for detecting the adipogenic, osteogenic and chondrogenic potentials of BSMCs, respectively. (E) Flow cytometry for evaluating BMSC surface markers. BMSCs, bone marrow mesenchymal stem cells.

The morphology of BMSCs was identified. As shown in Figure 1A, the cells at passage three were adherent and spindle‐shaped. Oil red O staining revealed the formation of lipid droplets in the cells after adipogenic differentiation induction (Figure 1B). Similarly, Alizarin red S staining and Alcian blue staining depicted that BMSCs retained the ability to differentiate into osteoblasts and chondrocytes, respectively (Figure 1C,D). Moreover, flow cytometry was conducted to detect the molecular markers of BMSCs. As shown by the results, the cells were significantly positive for the MSC markers CD44 and CD90, and were negative for the hematopoietic stem cell markers CD34 and CD11b (Figure 1E). Collectively, the above results confirmed the MSC properties of cultured BMSCs.

### 3.2 Overexpression of miR‐486 in BMSCs suppresses TGF‐β1‐triggered fibrosis in UFs

**FIGURE 2:**
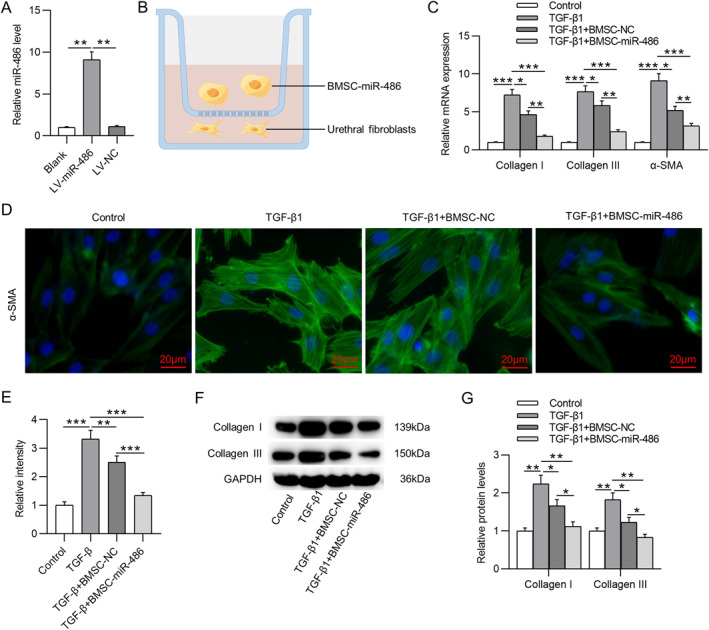
Overexpression of miR‐486 in BMSCs suppresses TGF‐β1‐triggered fibrosis in UFs. (A) RT‐qPCR for evaluating miR‐486 overexpression efficiency in BMSCs. (B) Schematic of the Transwell coculture system used in this study. (C) RT‐qPCR for detecting mRNA levels of collagen I/III and α‐SMA in UFs with indicated treatments. (D) IF staining for detecting α‐SMA expression (green) in UFs. (E) Quantitative results of IF staining. (F, G) Western blotting for measuring protein levels of collagen I and collagen III in UFs. *p ˂ 0.05, **p ˂ 0.01, ***p ˂ 0.001. BMSCs, bone marrow mesenchymal stem cells; IF, immunofluorescence; RT‐qPCR, real time quantitative polymerase chain reaction; TGF, transforming growth factor; UFs, urethral fibroblasts; α‐SMA, alpha smooth muscle actin.

For stable overexpression of miR‐486 in BMSCs, LV‐miR‐486 was transduced into BMSCs, and the overexpression efficiency was confirmed by RT‐qPCR analysis (Figure 2A). Then, to detect the effect of miR‐486‐overexpressed BMSCs on the fibrotic phenotype of UFs, we cocultured BMSC‐NC or BMSC‐miR‐486 with UFs in the presence of TGF‐β1 (Figure 2B). Subsequently, we measured the expression of fibrosis‐related genes in UFs with different treatments. As expected, TGF‐β1 treatment prominently increased mRNA levels of collagen I, collagen III, and α‐SMA in UFs (Figure 2C), confirming the promotion effect of TGF‐β1 on fibrosis. Moreover, BMSC‐NC or BMSC‐miR‐486 markedly attenuated TGF‐β1‐triggered increase in mRNA levels of fibrosis‐related genes. Additionally, in terms of decreasing collagen I/III and α‐SMA levels in TGF‐β1‐treated UFs, BMSC‐miR‐486 was much more effective than BMSC‐NC (Figure 2C). The expression levels of these fibrotic markers in UFs under different treatments were further confirmed by IF staining (Figure 2D,E) as well as western blotting (Figure 2F,G). Taken together, BMSCs with miR‐486 overexpression could attenuate TGF‐β1‐triggered fibrosis in UFs.

### 3.3 miR‐486 targets Col13a1

**FIGURE 3:**
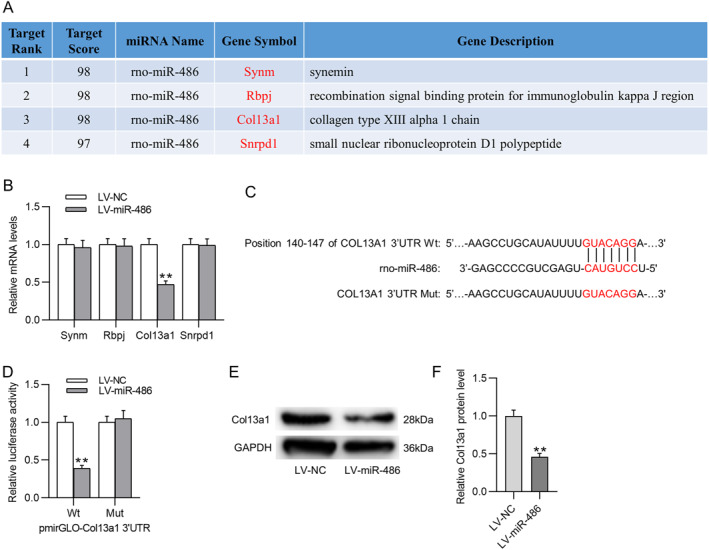
miR‐486 targets Col13a1. (A) Candidate target genes of miR‐486 predicted by miRDB database (screening condition: Target Score ˃96). (B) RT‐qPCR for detecting mRNA levels of predicted candidate genes in UFs with or without miR‐486 overexpression. (C) TargetScan database predicts the putative binding site between miR‐486 and Col13a1 3′UTR. (D) Luciferase reporter assay for verifying the binding relation between miR‐486 and Col13a1. (E, F) Western blotting for evaluating Col13a1 protein expression in UFs with or without miR‐486 upregulation. **p ˂ 0.01. 3′UTR, 3′ untranslated regions; RT‐qPCR, real time quantitative polymerase chain reaction; UFs, urethral fibroblasts.

To explore the potential mechanism of miR‐486 in regulating TGF‐β1‐induced urethral fibrosis, miRDB database was utilized to predict miR‐486 downstream target genes. As displayed in Figure 3A, four candidate genes were screened out with the screening condition of Target Score ˃96. Among these four genes, only Col13a1 was observed to be significantly downregulated in miR‐486‐overexpressed UFs (Figure 3B). Therefore, Col13a1 was selected for further analysis. TargetScan database predicts the complementary site between miR‐486 and Col13a1 3′UTR (Figure 3C). Then we mutated the predicted binding site on Col13a1 3′UTR and performed the luciferase reporter assay to verify the binding relation between the two molecules. As revealed by the results, the luciferase activity of Col13a1 was markedly weakened by upregulation of miR‐486 in UFs but was not significantly changed after mutation (Figure 3D). Furthermore, Col13a1 protein expression was prominently reduced in LV‐miR‐486 treated UFs as compared to that in LV‐NC‐treated UFs (Figure 3E,F), indicating that miR‐486 might negatively regulate Col13a1 in UFs.

### 3.4 BMSC‐miR‐486 alleviates TGF‐β1‐triggered US in rats

**FIGURE 4:**
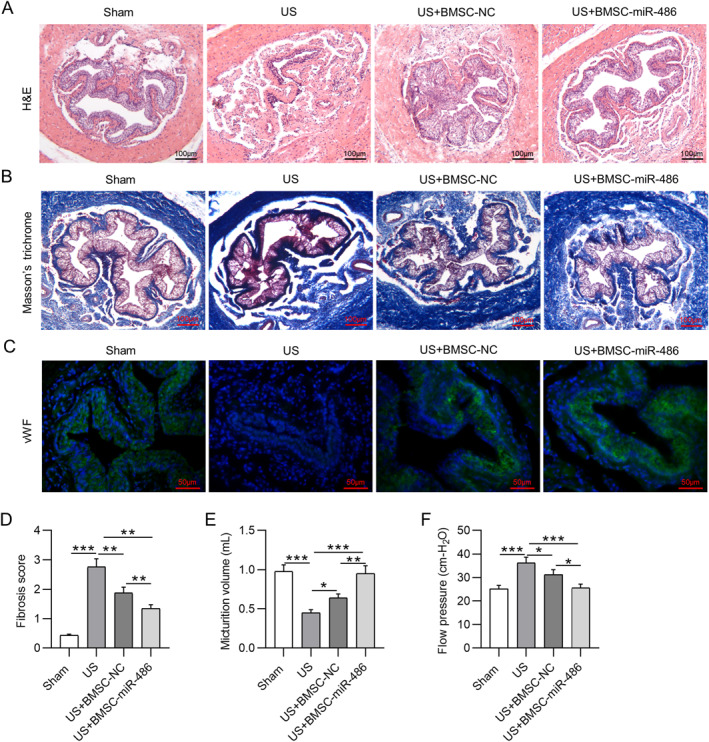
BMSC‐miR‐486 alleviates TGF‐β1‐triggered US in rats. (A) Representative images of H&E staining for observing the histopathological changes in midshaft sections of rat penises. (B) Representative images of Masson's trichrome staining for detecting collagen deposition (blue) in the urethral tissue of each group. (C) IF staining of vWF (green) for observing the formation of epithelium in rat urethra. (D) Fibrosis score of each group from Masson's trichrome staining. (E, F) Evaluation of the urodynamic parameters micturition volume (E) and flow pressure (F) in each group. *p ˂ 0.05, **p ˂ 0.01, ***p ˂ 0.001. BMSC, bone marrow mesenchymal stem cell; H&E, hematoxylin‐eosin; IF, immunofluorescence; TGF, transforming growth factor; US, urethral stricture; vWF, Von Willebrand Factor.

We further assessed the in vivo roles of BMSC‐miR‐486 in a rat US model. Histologic analysis was performed to evaluate the pathological changes in midshaft sections of rat penises. As depicted by H&E staining, relative to the sham‐operated rats, TGF‐β1‐induced US rats exhibited a significantly narrow urethral lumen (Figure 4A). Masson's trichrome staining revealed obvious submucosal fibrosis with dense collagen bundles in the urethral tissue of US rats (Figure 4B,D). However, US rats with administration of BMSC‐miR‐486 showed mild submucosal fibrosis and fewer collagen bundle depositions (Figure 4A,B,D). Furthermore, IF staining of vWF showed that the epithelium and the muscle layer were not fully formed in the urethra of US group, while BMSC‐miR‐486 significantly improved the formation of the epithelium and the muscle layer in the urethra of US rats (Figure 4C). Moreover, we measured the urodynamic parameters of rats in each group. As shown in Figure 4E, the micturition volume was significantly decreased in US rats compared to sham‐operated rats, whereas transplantation of BMSC‐miR‐486 significantly increased the micturition volume in US rats. In comparison to sham‐operated rats, US rats exhibited a significant increase in the flow pressure (Figure 4F). However, BMSC‐miR‐486 reduced the flow pressure in US rats (Figure 4F). These results suggested that overexpression of miR‐486 in BMSCs alleviated TGF‐β1‐triggered functional and pathological changes in the urethra of US rats.

### 3.5 Overexpression of miR‐486 in BMSCs alleviates urethral fibrosis in US rats

**FIGURE 5:**
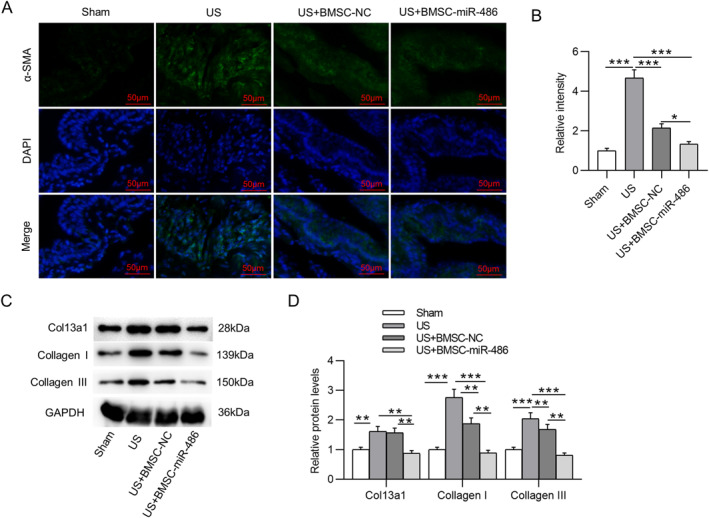
Overexpression of miR‐486 in BMSCs alleviates urethral fibrosis in US rats. (A) IF staining for detecting α‐SMA expression in the urethral tissue of rats in each group. (B) Quantitative results of IF staining. (C, D) Western blotting for measuring collagen I/III and Col13a1 protein levels in the penile midshaft tissue of rats. *p ˂ 0.05, **p ˂ 0.01, ***p ˂ 0.001. BMSCs, bone marrow mesenchymal stem cells; IF, immunofluorescence; US, urethral stricture; α‐SMA, alpha smooth muscle actin.

We then estimated the expression levels of fibrotic markers in the urethral tissue of each group. Notably, relative to that in the US group, α‐SMA expression was markedly decreased in the US + BMSC‐miR‐486 group (Figure 5A,B). Similarly, collagen I and collagen III protein levels in US rats were much higher than those in the sham‐operated rats, while BMSC‐NC or BMSC‐miR‐486 partially reversed the elevation of collagen I and collagen III protein levels in US rats. Importantly, BMSC‐miR‐486 exerted a more significant alleviative effect on collagen deposition than BMSC‐NC (Figure 5C,D). Additionally, Col13a1 protein expression in the urethra of US rats was prominently elevated but was decreased upon BMSC‐miR‐486 treatment (Figure 5C,D).

### 3.6 miR‐486 negatively regulates Col13a1 in the urethra of US rats

**FIGURE 6:**
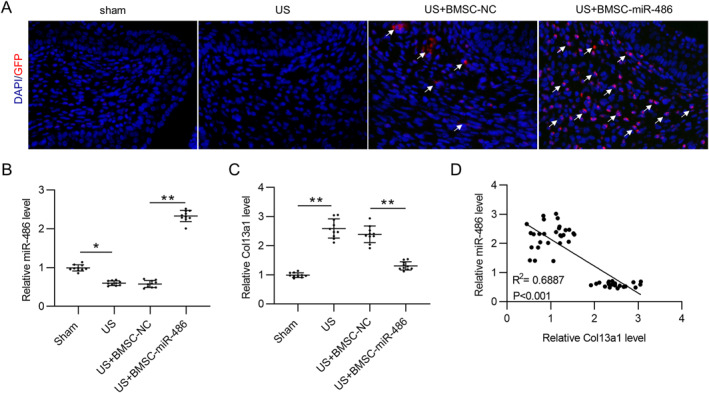
miR‐486 negatively regulates Col13a1 in the urethra of US rats. (A) Representative images showing the distribution of transplanted BMSCs (GFP‐labeled) in rat urethra. (B, C) RT‐qPCR for detecting miR‐486 (B) and Col13a1 (C) expression in the penile midshaft tissue of each group. *N* = 10 rats/group. (D) Pearson's correlation analysis for evaluating the expression correlation between miR‐486 and Col13a1 in the urethra of US rats. *N* = 30. *p ˂ 0.05, **p ˂ 0.01. BMSCs, bone marrow mesenchymal stem cells; RT‐qPCR, real time quantitative polymerase chain reaction; US, urethral stricture.

As shown in Figure 6A, GFP‐labeled BMSCs were detectable in the midshaft sections of rat penises in US + BMSC‐NC and US + BMSC‐miR‐486 groups. In addition, the fluorescence intensity was significantly higher in BMSC‐miR‐486 group than BMSC‐NC group (Figure 6A). Subsequently, we examined the expression levels of miR‐486 and Col13a1 in the urethral tissue of each group. As shown in Figure 6B, as compared to the sham group, miR‐486 level in the US group was markedly reduced but was significantly enhanced in the US + BMSC‐miR‐486 group, confirming the successful overexpression of miR‐486 in the urethra of US rats. On the contrary, Col13a1 expression in the US group was significantly higher than that in the sham group, whereas this effect was attenuated by BMSC‐miR‐486 administration (Figure 6C). In addition, no significant difference was observed in miR‐486 and Col13a1 expression between the US group and the US + BMSC‐NC group (Figure 6B,C). Pearson's correlation analysis revealed the negative correlation between miR‐486 expression and Col13a1 expression in the urethra of US rats (Figure 6D).

## 4 DISCUSSION

The present study revealed that overexpression of miR‐486 in BMSCs alleviated TGF‐β1‐triggered fibrosis in UFs. Moreover, BMSC‐miR‐486 ameliorated urethral narrowing and collagen deposition in the urethra of US rats. Mechanistically, miR‐486 targeted and negatively regulated Col13a1 in the urethral tissue of US rats.

Fibrotic scar formation within the urethral lumen after injury is closely associated with the pathogenesis of US, especially recurrent US.^30^ Several anti‐fibrotic drugs such as mitomycin C and halofuginone, have been used to limit the recurrence of US after endoscopic management; however, none of these agents exhibit sufficient efficacy after administration.^31^ Thus, it is imperative to further understand the mechanism underlying the pathogenesis of US and find more effective therapeutic approaches for this disease. Increasing evidences have suggested that MSCs exhibit great therapeutic effects on fibrotic diseases.^32^ Importantly, it was reported that local injection of BMSCs or their extracellular vehicles attenuated ureteral fibrosis in a rat model of ureteral stricture.^33^ Shi et al. demonstrated that BMSCs‐derived exosomes could protect against the formation of US in rats.^16^ These evidences indicate the potential therapeutic effects of BMSCs on the development of US. Moreover, genetic modification of MSCs has been increasingly studied to enhance the therapeutic effects of MSCs.^34^, ^35^ Herein, we examined the effects of miR‐486‐modified BMSCs on urethral fibrosis.

Many reports have indicated that miR‐486 functions as a protective miRNA in multiple diseases. For instance, overexpressing miR‐486 protects against cardiac ischemia/reperfusion injury and is essential for exercise‐triggered myocardial protection.^36^ Likewise, miR‐486 overexpression suppresses the expression of fibrotic genes including α‐SMA, PAI‐1, and TSP‐1 in cardiac myofibroblasts, consequently improving cardiac fibrosis and pathologic remodeling in myocardial infarction.^37^ Importantly, Lin et al. demonstrated that miR‐486 was prominently downregulated in the injured urethral specimens of rats as compared to that in the normal urethral specimens.^25^ Nonetheless, it is unclear whether miR‐486 affects the pathogenesis of US. Consistent with previous reports, our results revealed the therapeutic effect of BMSCs on urethral fibrosis in both TGF‐β1‐induced UFs and US rats, while overexpression of miR‐486 further augmented this effect. Similar to previous evidence,^37^ this study showed that miR‐486 suppressed α‐SMA expression. Moreover, it was found that overexpressing miR‐486 in BMSCs markedly blocked collagen deposition in TGF‐β1‐triggered UFs and the urethral tissue of US rats. These results indicated that miR‐486 overexpression in BMSCs attenuated urethral fibrosis induced by TGF‐β1. Additionally, our study depicted that BMSC‐miR‐486 could improve the formation of epithelium after injury and affect urodynamic parameters in US rats. Of note, we cocultured BMSCs with UFs in a Transwell system in vitro, which allowed the passage of molecules with a diameter ˂0.4 μm. Thus, miR‐486 may cause the above effects by inducing soluble factors or modifying exosomes derived from BMSCs. Furthermore, in vivo experiments showed that there were more GFP‐labeled BMSCs in the rat urethra in the miR‐486‐treated group than in the NC group, suggesting that overexpressing miR‐486 may enhance the engraftment and survival of transplanted BMSCs into the injury area.

It is recognized that miRNAs can bind to the 3′UTRs of targeted mRNAs via base pairing.^38^ Many mRNAs have been reported to be targeted by miR‐486, such as FOXD3, SRSF3, and CADM1.^37^, ^39^, ^40^ Here, by combining bioinformatics analysis and experimental validation, Col13a1 was verified to be a target of miR‐486. The Col13a1 gene is located at chromosome 10q22.1 and encodes the alpha chain of a nonfibrillar collagen that is localized to the plasma membrane.^41^ Previous evidence has suggested that Col13a1 upregulation is related to a high risk of disease progression in human urothelial carcinoma.^42^ Col13a1 plays a pivotal role in the synaptic maturation of neuromuscular junctions, and its mutation has been proposed as a new cause of congenital myasthenic syndromes.^43^ However, to our knowledge, its role in US remains unanswered. Our study revealed the upregulation of Col13a1 in the urethra of US rats, indicating its potential role in US progression. Additionally, the results displayed that Col13a1 was negatively regulated by miR‐486 in the urethra of US rat models. Further investigations are required in the future to elucidate Col13a1 functions in the pathogenesis of US.

In conclusion, this study reveals that miR‐486 modification can enhance the therapeutic potential of BMSCs in ameliorating TGF‐β1‐triggered urethral fibrosis and pathological damages in the fibroblasts and US rats. Additionally, miR‐486 targets and negatively regulates Col13a1 in US rats. Our findings may provide a basis for translating this treatment strategy into clinics to limit US recurrence after urethrotomy. In addition, future studies are needed to further elucidate the findings.

## AUTHOR CONTRIBUTIONS

Yali Xu and Lihong Huang were the main designers of this study. Yali Xu, Lihong Huang, Zhixin Qiu, Jiaqi Zhang, Xueyi Xue and Junshan Lin performed the experiments and analyzed the data. Yali Xu, Lihong Huang, Zhixin Qiu, Jiaqi Zhang, Xueyi Xue and Junshan Lin drafted the manuscript. All authors read and approved the final manuscript.

## CONFLICT OF INTEREST STATEMENT

The authors have no competing interests.

## ETHICS STATEMENT

All animal experiments were approved by the Ethics Committee of The First Affiliated Hospital, Fujian Medical University.

## RESEARCH INVOLVING HUMAN PARTICIPANTS AND/OR ANIMALS

All animal experiments were conducted following the NIH Guide for the Care and Use of Laboratory Animals and approved by the Ethics Committee of The First Affiliated Hospital, Fujian Medical University.

## INFORMED CONSENT

Not applicable.

## Data Availability

The datasets used or analyzed during the current study are available from the corresponding author on reasonable request.
